# Identification of a complex intrachromosomal inverted insertion in the long arm of chromosome 9 as a cause of tuberous sclerosis complex in a Korean family

**DOI:** 10.1002/mgg3.2330

**Published:** 2024-01-24

**Authors:** Seung Woo Ryu, Ji‐Hee Yoon, Dong‐wook Kim, Beomman Han, Heonjong Han, Joohyun Han, Hane Lee, Go Hun Seo, Beom Hee Lee

**Affiliations:** ^1^ 3billion, Inc Seoul South Korea; ^2^ Department of Pediatrics, Asan Medical Center Children's Hospital University of Ulsan College of Medicine Seoul Republic of Korea

**Keywords:** complex inversion, complex structural variant, genome sequencing, tuberous sclerosis complex

## Abstract

**Background:**

Tuberous sclerosis complex (TSC) is an autosomal dominant multisystem disorder, caused by a loss‐of‐function of either *TSC1* or *TSC2* gene. However, in 10%–15% TSC patients there is no pathogenic variant identified in either *TSC1* or *TSC2* genes based on standard clinical testing.

**Methods:**

In this study, genome sequencing was performed for families with clinical diagnosis of TSC with negative results from *TSC1* and *TSC2* single‐gene tests.

**Results:**

Herein, we report a family presenting a classical TSC phenotype with an unusual, complex structural variant involving the *TSC1* gene: an intrachromosomal inverted insertion in the long arm of chromosome 9. We speculate that the inverted 9q33.3q34.13 region was inserted into the q31.2 region with the 3′‐end of the breakpoint of the inversion being located within the *TSC1* gene, resulting in premature termination of *TSC1*.

**Conclusions:**

In this study, we demonstrate the utility of genome sequencing for the identification of complex chromosomal rearrangement. Because the breakpoints are located within the deep intronic/intergenic region, this copy‐neutral variant was missed by the *TSC1* and *TSC2* single‐gene tests and contributed to an unknown etiology. Together, this finding suggests that complex structural variants may be underestimated causes for the etiology of TSC.

## Graphical abstract

We report a family presenting classical tuberous sclerosis phenotype with an unusual, complex structural variant involving the *TSC1* gene: an intrachromosomal inverted insertion in the long arm of chromosome 9.

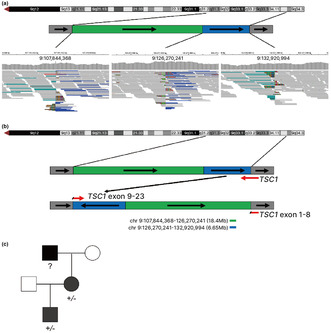

## 1 INTRODUCTION

Tuberous sclerosis complex (TSC) is an autosomal dominant multisystemic disorder, caused by loss‐of‐function of either the *TSC1* gene (OMIM: 191100) or the *TSC2* gene (OMIM: 191092) (Crino et al., 2006; Curatolo et al., 2008; Northrup et al., 1993). TSC is characterized by the formation of multiple benign tumors known as hamartoma throughout the affected areas of the body. In addition, other TSC‐related tumors such as subependymal giant cell astrocytomas (SEGAs), subependymal nodules (SENs), ungual fibromas (UFs), and lymphangioleiomyomatosis (LAM) can also be found (Klonowska et al., 2023; Northrup et al., 1993, 2021). Seizures, intellectual disability, and renal hemorrhage are also commonly observed (Northrup et al., 1993, 2021). The prevalence of TSC is estimated to be around 1:6760 to 1:13,520 live births (Ebrahimi‐Fakhari et al., 2018).

Diagnosis of TSC can be made both clinically and genetically. Clinical diagnosis can be made when at least two major clinical features of TSC such as ‘the presence of angiofibroma on facial skin’ and ‘two or more angiomyolipomas’ are observed or by the combination of one major and two minor features such as ‘presence of “confetti” skin lesions’ or ‘multiple renal cysts’ are observed (Northrup et al., 1993, 2021; Portocarrero et al., 2018). Genetic diagnosis of TSC can be established independent of clinical findings as the genetic finding can provide surveillance opportunities before symptoms arise. With proper surveillance, TSC can be medically managed and most fetal findings could be avoided (Crino et al., 2006; Curatolo et al., 2008; Sudarshan et al., 2021). Genetic diagnosis of TSC is made by the detection of a pathogenic variant within *TSC1* or *TSC2* genes. With panel testing using next‐generation sequencing (NGS), up to 85%–90% of suspected individuals are able to receive a genetic diagnosis. Of the genetically diagnosed cases, 10%–30% of the cases are found with variants in *TSC1* while the rest are diagnosed with variants in *TSC2* (Curatolo et al., 2008; Rosset et al., 2017). The majority of affected individuals carry premature terminating small nucleotide variants (SNV) or small indels (INDELS), such as nonsense, essential splice site, or frameshift variants (Rosset et al., 2017). Larger structural variants have also been described, with most of them being exonic deletions, contiguous gene deletion, and large intragenic or intergenic duplications (Brook‐Carter et al., 1994; Mongrain et al., 2020; Northrup et al., 2021; Sancak et al., 2005). More complex structural variants are extremely rare for TSC with only one inversion reported so far involving *TSC2* and *ABCA3* in an amniotic fluid sample (Klonowska et al., 2023).

In this report, we present a family with a classic TSC phenotype and an unusual, complex structural variant involving the *TSC1* gene. Both the patient and her son carried an intrachromosomal inverted insertion in the long arm of chromosome 9. This is by far the first description of a patient with intrachromosomal inverted insertion that leads to TSC.

## 2 MATERIALS AND METHODS

### 2.1 Editorial Policies and Ethical Considerations

The study was approved by an ethics committee by the ‘Korea National Institute of Health (KNIH)’ research project (project No.2021ER040200).

### 2.2 Participants

The patient was a 56‐year‐old Korean female born of a non‐consanguineous marriage with a clinical diagnosis of TSC. The patient and her 31‐year‐old similarly affected son were enrolled for a genome sequencing (GS) supported by the ‘Korea National Institute of Health (KNIH)’ research project (project No.2021ER040200). Informed consent for genetic testing was obtained from both participants.

### 2.3 Genome sequencing and data analysis

Genome sequencing was performed at Macrogen, Inc using high molecular weight genomic DNA extracted from whole blood samples. Illumina NovaSeq 6000 system (San Diego, CA, USA) was used for sequencing as 150 bp paired‐end reads. Sequencing data analysis was performed at 3billion, Inc. Alignment to the GRCh38 human reference genome was done using BWA‐MEM2, and samtools v1.15 was used for bam file sorting and marking duplicates (Li et al., 2009; Vasimuddin et al., 2019). Recalibration and variant calling for single nucleotide variants (SNVs) and small insertion/deletion variants (indels) were performed using GATK v4.2.14 (van der Auwera & O'Connor, 2020) Structural variants (SVs) were called using MANTA v1.6.0 (Chen et al., 2016), and 3bCNV, an internally developed tool (manuscript in preparation). Variants were annotated, filtered, and classified using EVIDENCE v3.2 which incorporates Ensembl Variant Effect Predictor (VEP) for annotation and the American College of Medical Genetics and Genomics (ACMG) guideline for classification (Richards et al., 2015; Seo et al., 2020). The filtered and classified variants were manually reviewed by medical geneticists and physicians. The most likely variants that can explain the patient's phenotype were selected for reporting.

## 3 RESULTS

### 3.1 Clinical information

**FIGURE 1:**
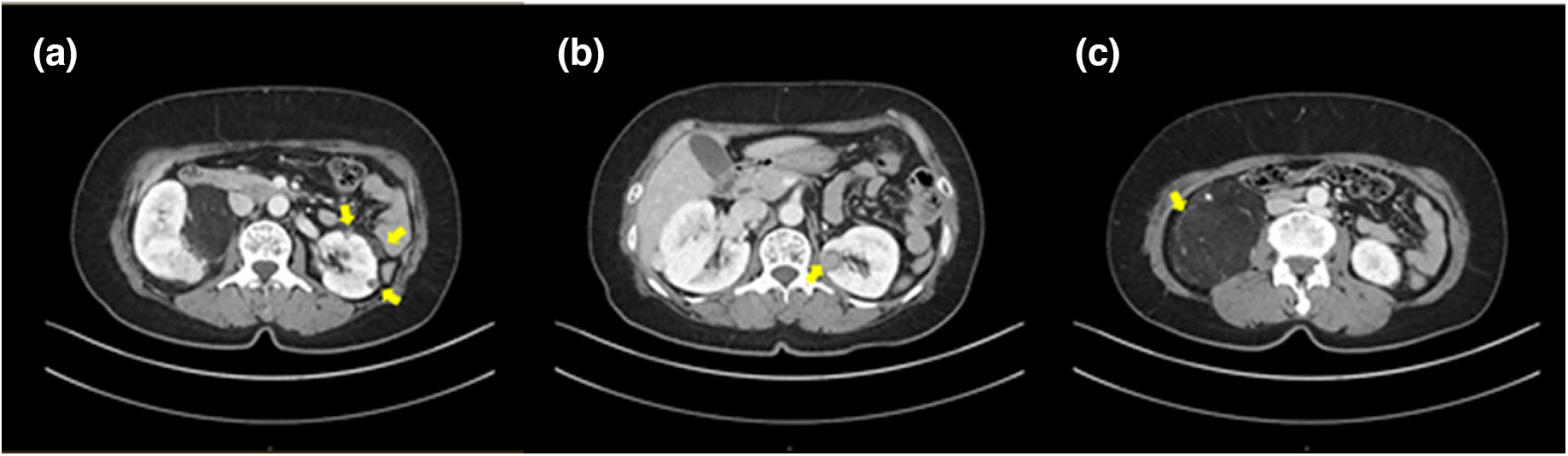
Abdominal and pelvic CT scan showing multiple angiomyolipomas. (a) Three small fatty masses in the left kidney. (b) Indeterminate enhancing mass, suggestive of angiomyolipoma with minimal fat in the left kidney. (c) An 8.5 cm fatty mass in the right kidney causing mild pelviectasis.

A 56‐year‐old Korean woman visited a urological outpatient clinic after a medical examination revealed bilateral renal masses. Abdominal and pelvic computed tomography (CT) scans showed multiple nodules in both kidneys, including an 8.5 cm fatty mass in the lower pole of the right kidney, suggestive of angiomyolipomas (Figure 1). The patient received selective embolization of the tumor feeders. She was referred to the medical genetics center for the possibility of a genetic condition. Physical examination revealed facial angiofibromas and periungual fibromas. The patient had no history of seizures and showed normal intelligence. Additionally, the patient had multiple family members, including her father, with facial angiofibromas. Furthermore, her son also presented facial angiofibromas and periungual fibromas. Further investigations revealed the presence of tuber and subependymal nodules on the brain MRI, as well as multifocal micronodular pneumocyte hyperplasia and multiple sclerotic bone lesions in the thoracic spine and ribs on the chest CT. Echocardiography did not detect any cardiac rhabdomyoma. No clinically significant SNVs/small indel variants were found in the coding regions of *TSC1* and *TSC2* genes by single‐gene tests. Moreover, no exon deletions were detected by the Multiplex Ligation‐dependent Probe Amplification (MLPA) analysis.

### 3.2 Identification of the disease‐causing genomic variant

**FIGURE 2:**
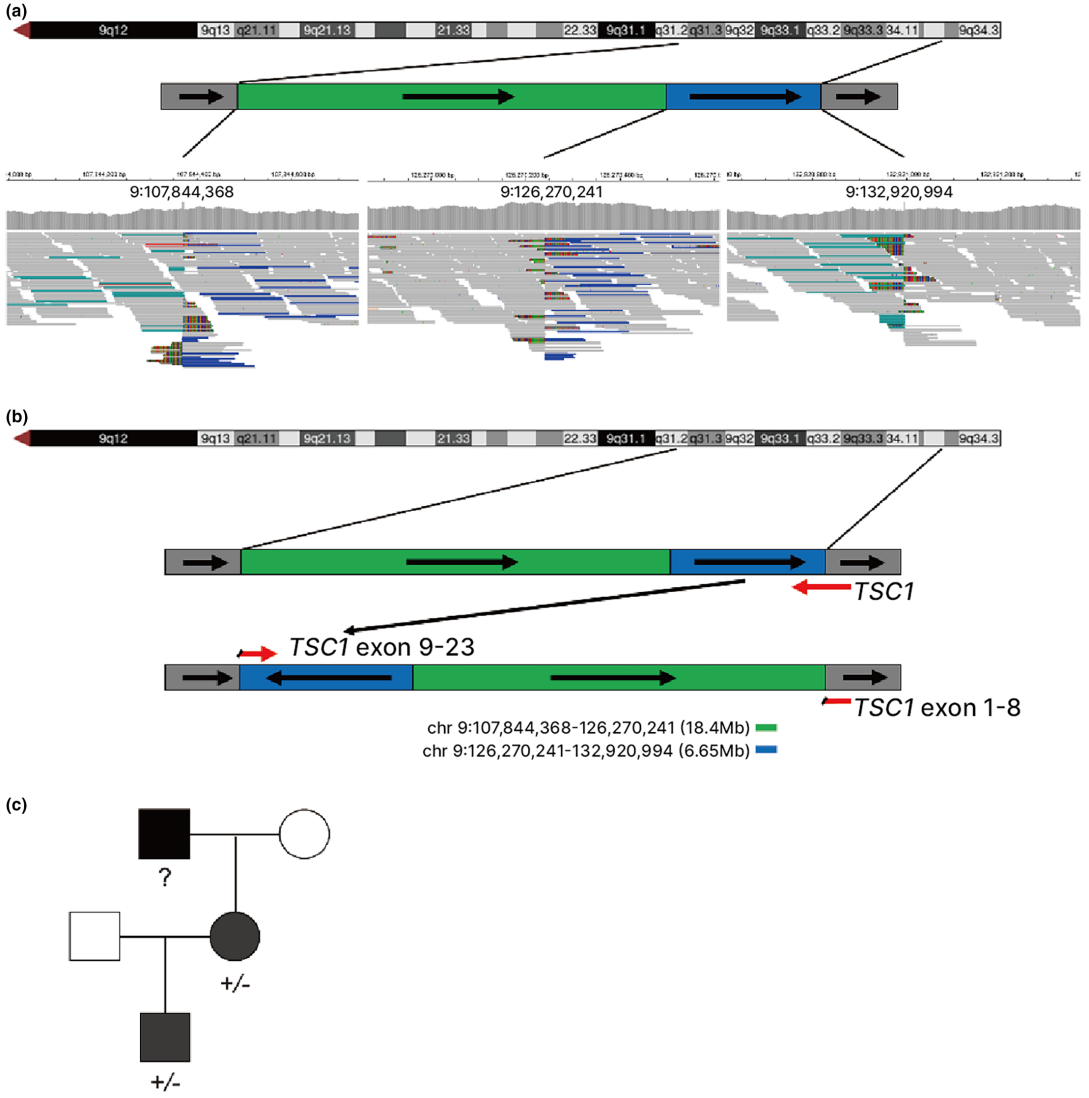
Intrachromosomal inverted insertion. (a) Integrative Genomics Viewer (IGV) view of the variant showing reads with anomalous read‐pair orientation, suggestive of an inverted insertion. (b) The top panel shows chromosome 9q cytoband, and the bottom two panels show the schematic diagram of the variant: the blue segment is the inverted region that is inserted into the 5′‐flanking region of the green segment. *TSC1* (NM_000368.5 transcript) gene is represented by a red arrow reflecting the transcription direction. (c) Segregation of the structural variant: similarly affected mother and son both carry the same intrachromosomal inverted insertion.

From genome sequencing, no clinically significant SNV/INDELs in the *TSC1*/*TSC2* genes or any other known disease genes were identified. However, two structural variants (SV) spanning *TSC1* were called by MANTA: a deletion at NC_000009.12:g.126,270,241‐132,920,994 and an inversion at NC_000009.12:g.107,844,368‐126,270,241 (Figure 2a). Further inspection of the variant revealed that the variant is an intrachromosomal inverted insertion for which the 9q33.3q34.13 (NC_000009.12:g.126,270,241‐132,920,994) region is invertedly inserted to the 9q31.2 (NC_000009.12:g.107,844,368‐107,844,369) position (Figure 2b). The 3′ breakpoint of the inversion was located in intron 8 (NC_000009.12.g:132,920,994‐132,920,995) of the *TSC1* gene. The effect of this variant is predicted to be similar to exon 9–23 deletion of *TSC1* (NM_000368.5 transcript) although downstream functional analysis would be required to confirm the true effect of the variant. Both variants were also called in the patient's son, suggesting that the two variants are *in cis* and therefore these variants are most likely a single heterozygous variant. (Figure 2c).

## 4 DISCUSSION

TSC (OMIM: 605284), caused by rare loss‐of‐function variants in the *TSC1* and *TSC2* genes, is a complex disorder for which there are interventional treatments that often require lifelong surveillance for the manifestation of tumors (Northrup et al., 2021; Zöllner et al., 2020). Newborn screening has successfully begun to provide an early diagnosis, even before the presentation of symptoms (Qiao et al., 2021; Su et al., 2021). However, in 10%–15% TSC patients there is no pathogenic variant identified in either *TSC1* or *TSC2* genes based on standard clinical testing and are generally attributed to low mosaicism, deep intronic/promoter variants, and additional causative gene loci yet to be identified (Brook‐Carter et al., 1994; Curatolo et al., 2008; Klonowska et al., 2023; Sancak et al., 2005; Ye et al., 2022). For instance, in one study, undetected somatic mosaic variants were the most common cause for undiagnosed patients with TSC with up to 51.6% (16/31) of undiagnosed patients being found to carry a disease‐causing mosaic variant (Ye et al., 2022). Our study suggests that complex structural variants may also be overlooked by genomic events underlying TSC.

In this report, we described a patient and her son with TSC who carries an unusual chromosomal rearrangement involving *TSC1*. Because the breakpoints of the rearrangement are located within the deep intronic/intergenic region, this copy‐neutral variant was not detected by single gene tests using conventional Sanger sequencing and MLPA methods that read the protein‐coding regions, leaving the patients with an unknown genetic etiology. Panel testing or exome sequencing would have missed the variant as well. Our hypothesis is that the similarly affected father carries the same variant and one of the patient's unaffected paternal grandparents might have carried a balanced paracentric inverted chromosome 9 with the breakpoints falling outside the *TSC1* gene. (Figure 2a) From a homologous recombination crossover event with normal chromosome 9, the patient's father must have received a chromosome with the inverted chromosome 9q33.3q34.13 segment inserted into 9q34.3. However, the proband's father or parental grandparents' samples are not available and the mechanism remains elusive.

Detection of large complex rearrangement is commonly missed in clinical laboratories even with GS as they remain a challenge to be called by bioinformatics tools. Balanced chromosomal rearrangements are especially difficult to detect as they are copy‐number neutral. Polymorphic inversions could be harmless and have no phenotypic consequences (Puig et al., 2015). For example, inverted chromosome 9 is considered clinically benign except for causing infertility due to homologous crossover events during meiosis (Ferfouri et al., 2009; Hsu et al., 1987; Puig et al., 2015; Thomas et al., 2008). If such variants are common in the population, they would be classified as likely benign/benign. However, the interpretation of a de novo or rare inversion is challenging unless the breakpoint(s) of the inversion falls within a known disease gene and is predicted to disrupt the gene function as it did in the patients reported here. Inversion with intergenic breakpoints could still cause disease by altering the expression of a nearby gene. However, additional functional studies such as transcriptome sequencing would be needed to prove that gene expressions are perturbed (Anger et al., 2014; Brown et al., 2010; Jones et al., 2013, p. 5; Utami et al., 2014).

Upon receiving a genetic diagnosis, the patient and her son are being followed up with annual examinations for the TSC, including brain MRI and chest/abdominal CT. Appropriate family planning consultation would also be available for the affected son and other unaffected family members who may carry the benign inversion. Structural variants are estimated to account for about 6%–9% of the *TSC1*/*TSC2* patients (Jang et al., 2012; Karczewski et al., 2020; Rosset et al., 2017; Sancak et al., 2005). For example, larger gene deletion syndrome such as ‘*TSC2* and *PKD1* deletion’ has been described as a contiguous gene deletion syndrome, ‘Polycystic kidney disease, infantile severe, with tuberous sclerosis (OMIM: 600273)’, as *PKD1* is in close proximity to *TSC2* (van den Ouweland et al., 2011). Other structural variants such as duplication and deletions of *TSC1* or *TSC2* have also been described in patients (Mongrain et al., 2020; Sancak et al., 2005), including one inversion described in an amniotic fluid sample (Klonowska et al., 2023). Therefore, this finding demonstrates that the utility of GS in deciphering complex structural variants is significant, and it is evident that GS should be considered when a clear clinical diagnosis is not supported by other conventional genetic testing.

## AUTHOR CONTRIBUTIONS

Go Hun Seo, Beom Hee Lee, and Hane Lee are responsible for study design and conceptualization, Seung Woo Ryu, Go Hun Seo, and Hane Lee are responsible for the evaluation of data, Dong‐Wook Kim, Beomman Han, Heonjong Han, and Joohyun Han are responsible for bioinformatics analysis, Ji‐Hee Yoon, Beom Hee Lee are responsible for clinical part of the manuscript, and Seung Woo Ryu and Ji‐Hee Yoon are responsible for the writing of the manuscript and finalized the manuscript. All authors read the manuscript and contributed intellectual content.

## FUNDING INFORMATION

The study was supported by the ‘Korea National Institute of Health (KNIH)’ research project (project No.2021ER040200).

## CONFLICT OF INTEREST STATEMENT

The authors declare no conflict of interest. The funders had no role in the design of the study; in the collection, analysis, or interpretation of the data; in the writing of the manuscript, or in the decision to publish the results.

## Data Availability

The data presented in this study are available upon request from the corresponding author. The data are not publicly available due to restrictions of patient privacy.
